# An integrated approach to understand apicomplexan metabolism from their genomes

**DOI:** 10.1186/1471-2105-15-S3-A3

**Published:** 2014-02-11

**Authors:** Achchuthan Shanmugasundram, Faviel F Gonzalez-Galarza, Jonathan M Wastling, Olga Vasieva, Andrew R Jones

**Affiliations:** 1 Institute of Integrative Biology, University of Liverpool, Biosciences Building, Crown Street, Liverpool L69 7ZB, UK; 2 Institute of Infection and Global Health, University of Liverpool, Liverpool Science Park Innovation Centre 2, 146 Brownlow Hill, Liverpool L3 5RF, UK

## Background

The *Apicomplexa* is a large phylum of intracellular parasites that show great diversity and adaptability in the various ecological niches they occupy. They are the causative agents of human and animal infections including malaria, toxoplasmosis and theileriosis, which have a huge economic and social impact. A number of apicomplexan genomes have been sequenced and are publicly available. However, the prediction of gene models and annotation of gene functions remains challenging.

## Methods

We have utilised an approach called ‘metabolic reconstruction’, in which genes are systematically assigned to functions within pathways/networks [1-4]. Functional annotation and metabolic reconstruction was carried out using a semi-automatic approach, integrating genomic information with biochemical evidence from the literature. The functions were automatically assigned using a sequence similarity-based approach and protein motif information. Experimental evidence was also accommodated in the confirmation of functions and the grouping of genes into metabolic pathways.

## Results

A web database named Library of Apicomplexan Metabolic Pathways (LAMP, http://www.llamp.net) [5] was developed to deposit the reconstructed metabolic pathways of *Toxoplasma gondii*, *Neospora caninum*, *Cryptosporidium* and *Theileria* species and *Babesia bovis*. Each metabolic pathway page contains an interactive metabolic pathway map, gene annotations hyperlinked to external resources and detailed information about the metabolic capabilities. This analysis led to the identification of *missing enzymes* that must be present to complete the metabolic pathway and *orphan genes* (incorrect enzyme annotations or enzymes that are involved in salvage of metabolites) that are isolated in pathways that are otherwise absent. The compilation and annotation of metabolic pathways and the comparative analysis of the overall metabolic capabilities of apicomplexan species enabled identification of differences in their ability to synthesise or depend on hosts for several metabolites (Table 1, Additional file 1).

**Table 1:** A survey of the data available for the different apicomplexan genomes in LAMP. The analysis is updated from the survey table published in the previous publication [5]

Organism	No of metabolic pathways	No of unique enzymes^a^	No of missing enzymes^b^	No of reactions^c^	Total no of metabolites^d^	No of metabolites from host^e^	No of end metabolites to host or of unknown fate^f^
***T. gondii***	51	419	17	509	500	41	23
***N. caninum***	51	412	23	509	500	41	23
***C. muris***	31	224	15	255	281	32	7
***C. parvum***	28	207	10	231	261	31	8
***C. hominis***	28	200	17	230	261	31	8
***T. parva***	32	213	17	234	258	26	9
***T. annulata***	32	214	16	235	258	26	9
***B. bovis***	32	216	11	233	256	26	9

^a^ Unique Enzymes represent total unique enzyme activities (enzymes with full, partial and no EC numbers) annotated to be present in the pathways for an organism.

^b^ Missing enzymes represent the enzymes need to be present to complete the metabolic pathways. They may either be missing in the gene model predictions or may be absent in the organism.

^c^ Total number of biochemical reactions annotated to metabolic pathways from KEGG REACTION database.

^d^ Total number of metabolites annotated to metabolic pathways from KEGG COMPOUND and KEGG GLYCAN databases.

^e^ Number of precursor metabolites in the metabolic pathways that are annotated to be obtained from host. This number does not include any other metabolites that are obtained from host and not part of any of the annotated pathways in LAMP.

^f^ Number of end metabolites in the metabolic pathways that does not end up in a downstream pathway. These can either be metabolites that end up in host or the fate pathways are unknown.

## Conclusions

The carefully annotated metabolic pathways and the comparative analysis of metabolism for eight apicomplexan species are publicly available for the research community in the LAMP database (http://www.llamp.net). This has improved the functional annotation immensely and led to identification of putative drug targets. The hyperlinks for LAMP metabolic pathway annotations are available from the respective gene pages of the *T. gondii* primary database, ToxoDB (release 9) [6], enabling a wider reach for LAMP.

## Supplementary Material

Additional file 1 A colour-coded table for comparison of the presence and absence of metabolic capabilities in different apicomplexan species. The green colour denotes the presence and red colour denotes the absence of metabolic capabilities in the species. The capabilities are grouped under pathways as grouped in the maps available in LAMP. The metabolic pathways are in bold letters and the capabilities under a pathway are in regular letters. Although *Plasmodium falciparum* is not available in LAMP, it is provided for comparison. *Pfa* – *P. falciparum*, *Tgo* – *Toxoplasma gondii*, *Nca* – *Neospora caninum*, *Cmu* – *Cryptosporidium muris*, *Cpa* – *Cryptosporidium parvum*, *Cho* – *Cryptosporidium hominis*, *Bbov* – *Babesia bovis*, *Tpa* – *Theileria parva*, *Ta* – *Theileria annulata*. Click here for file
