# Supplementary figures and images for: An integrated approach to understand apicomplexan metabolism from their genomes
Source: BMC Bioinformatics. 2014 Feb 11;15(Suppl 3):A3. doi: 10.1186/1471-2105-15-S3-A3 (PMC4071867; doi:10.1186/1471-2105-15-S3-A3)

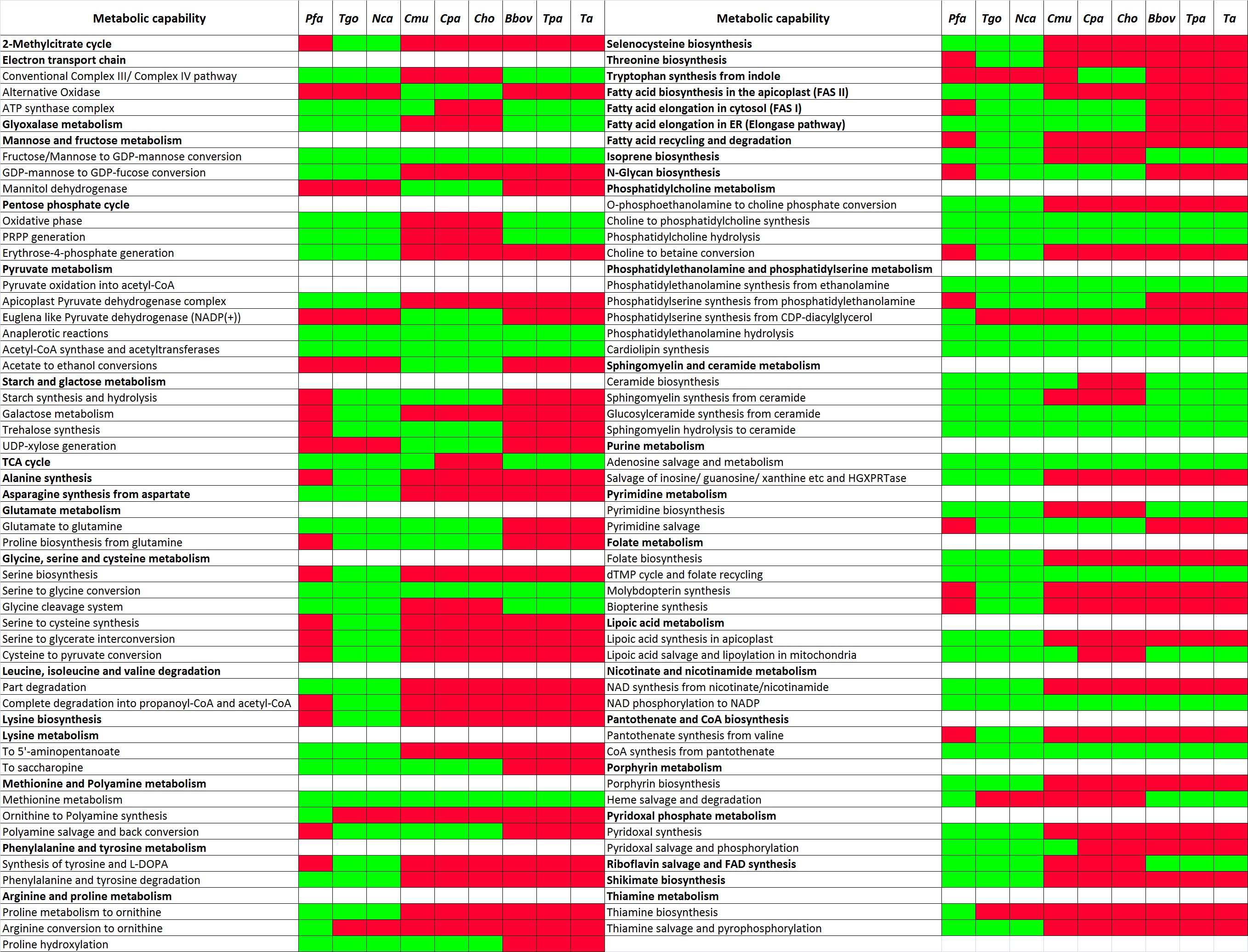

Supplement: Additional file 1 — A colour-coded table for comparison of the presence and absence of metabolic capabilities in different apicomplexan species. The green colour denotes the presence and red colour denotes the absence of metabolic capabilities in the species. The capabilities are grouped under pathways as grouped in the maps available in LAMP. The metabolic pathways are in bold letters and the capabilities under a pathway are in regular letters. Although Plasmodium falciparum is not available in LAMP, it is provided for comparison. Pfa – P. falciparum, Tgo – Toxoplasma gondii, Nca – Neospora caninum, Cmu – Cryptosporidium muris, Cpa – Cryptosporidium parvum, Cho – Cryptosporidium hominis, Bbov – Babesia bovis, Tpa – Theileria parva, Ta – Theileria annulata. [file 1471-2105-15-S3-A3-S1.tif]
